# Supplementary material for: Associations between Taste and Smell Sensitivity, Preference and Quality of Life in Healthy Aging—The NutriAct Family Study Examinations (NFSE) Cohort
Source: Nutrients. 2022 Mar 8;14(6):1141. doi: 10.3390/nu14061141 (PMC8950182; doi:10.3390/nu14061141)
Supplement: Supplementary file 1 [file nutrients-14-01141-s001.zip › nutrients-1613502-supplementary.pdf]

Taste quality and age and QoL

A linear mixed model is fitted to predict each taste sensitivity with age and gender as a random effect with varying slopes and intercepts.

For sour, conditional R^2=0.0658, marginal R^2=0.0045, β_0=0.112 (95% CI=[-0.119, 0.343], $t_{0.98}$=0.954, p=0.518), β_{age}=0.008 (95% CI=[-0.008, 0.024], $t_{223.6}$=1.003, p=0.317).

For salty, conditional R^2=0.0214, marginal R^2=0.00042, β_0=1.293 (95% CI=[1.155, 1.431], $t_{0.98}$=18.392, p=0.04), β_{age}=-0.0024 (95% CI=[-0.017, 0.012], $t_{208.7}$=-0.304, p=0.761).

For bitter, conditional R^2=0.0784, marginal R^2=0.00051, β_0=-1.135 (95% CI=[-1.465, -0.801], $t_{0.99}$=-6.745, p=0.096, β_{age}=0.0035 (95% CI=[-0.017, 0.024], $t_{219.7}$=0.338, p=0.736).

For sweet, conditional R^2=0.0149, marginal R^2=0.00075, β_0=1.289 (95% CI=[1.168, 1.411], $t_{0.93}$=20.79, p=0.04), β_{age}=-0.003 (95% CI=[-0.007, 0.011], $t_{196.8}$=0.408, p=0.683).

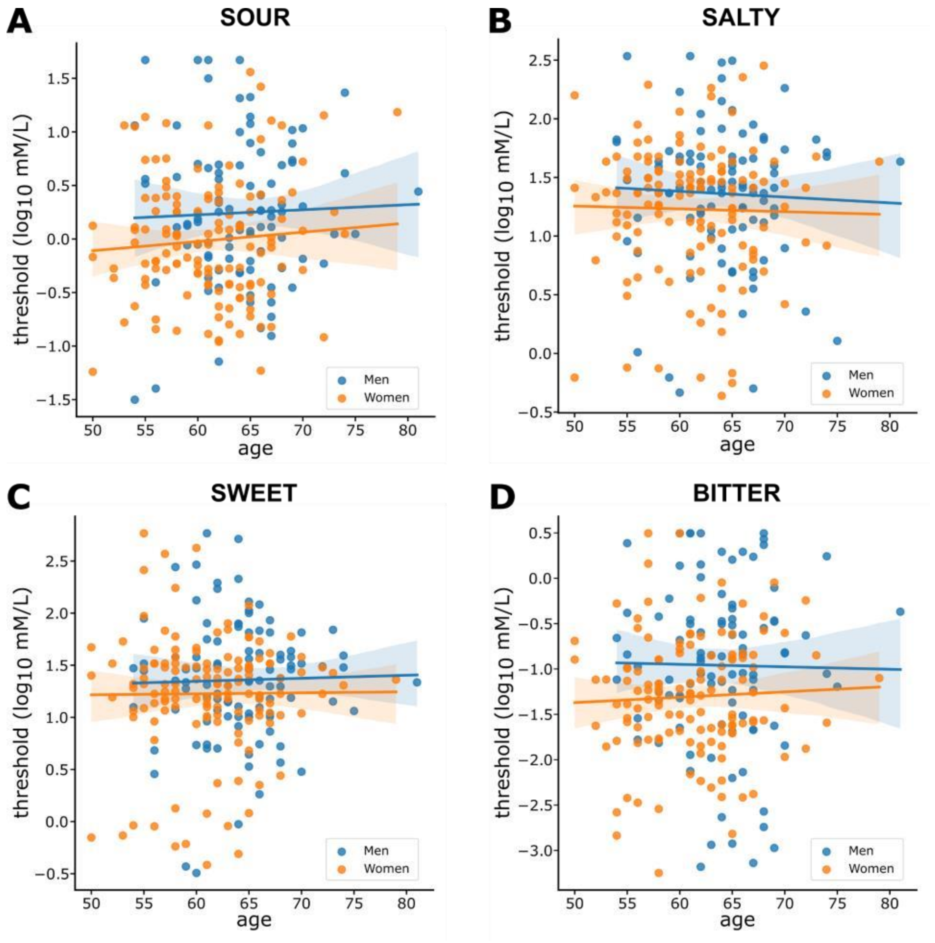

Supplementary Figure S1. Taste quality and age. Scatter plot with linear regression line of taste thresholds fitted across age for men and women separately. Shaded regions represent 95% CI. Each dot is individual data with a different color indicating gender.

A simple linear regression was fitted to test whether taste sensitivity can be predicted by QoL, separately for each taste and each component (mental and physical) of QoL. For the mental component of QoL, none of the taste sensitivity can be predicted by QoL (sour: R^2=0.0152, p=0.064; salty: R^2=0.0143, p=0.075; sweet: R^2=0.0061, p=0.236; bitter: R^2=0.0143, p=0.075). However, for the mental component of QoL, only sour and sweet taste sensitivity can be significantly predicted by QoL (sour: R^2=0.0218, p=0.026; salty: R^2=0.0031, p=0.399; sweet: R^2=0.0018, p=0.04; bitter: R^2=0.0020, p=0.512)

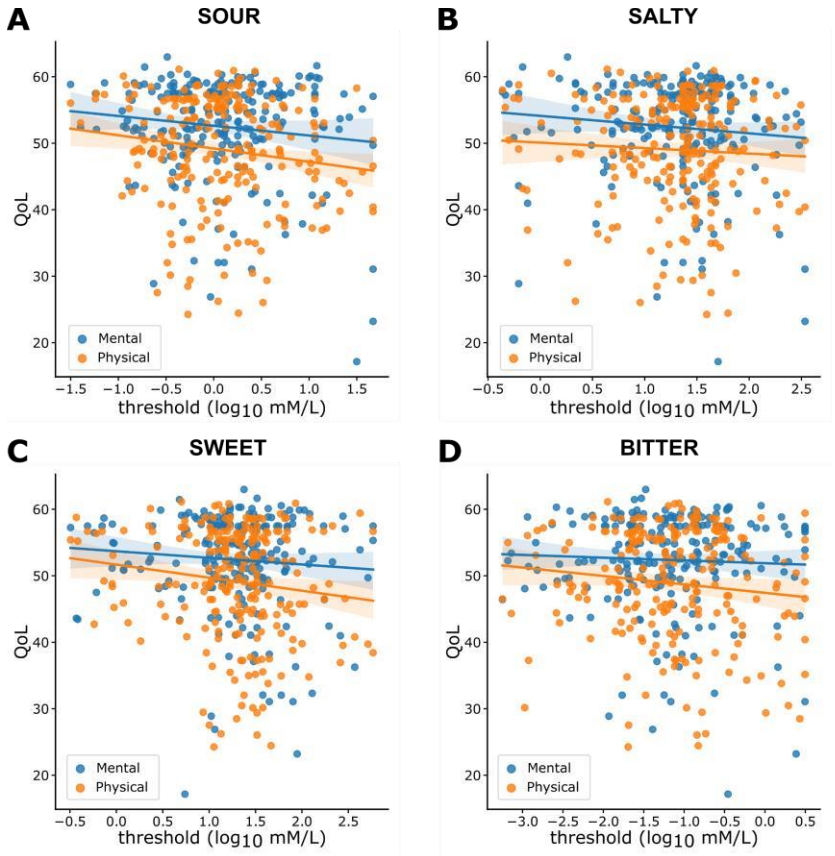

Supplementary Figure S2. Taste quality and QoL. Scatter plot with linear regression line of taste thresholds fitted across each component of QoL (mental and physical). Shaded regions represent 95% CI. Each dot is individual data with a different color indicating a component of QoL.
